# Emerging Concepts and Technologies in Vaccine Development

**DOI:** 10.3389/fimmu.2020.583077

**Published:** 2020-09-30

**Authors:** Morgan Brisse, Sophia M. Vrba, Natalie Kirk, Yuying Liang, Hinh Ly

**Affiliations:** ^1^ Biochemistry, Molecular Biology, and Biophysics Graduate Program, University of Minnesota Twin Cities, St. Paul, MN, United States; ^2^ Department of Veterinary & Biomedical Sciences, University of Minnesota Twin Cities, St. Paul, MN, United States; ^3^ Comparative Molecular Biosciences Graduate Program, University of Minnesota Twin Cities, St. Paul, MN, United States

**Keywords:** non-viral DNA-RNA vaccines, nanoparticle vaccines, virus-like particle vaccines, cancer vaccines, substance abuse, noncommunicable disease, infectious disease, COVID19

## Abstract

Despite the success of vaccination to greatly mitigate or eliminate threat of diseases caused by pathogens, there are still known diseases and emerging pathogens for which the development of successful vaccines against them is inherently difficult. In addition, vaccine development for people with compromised immunity and other pre-existing medical conditions has remained a major challenge. Besides the traditional inactivated or live attenuated, virus-vectored and subunit vaccines, emerging non-viral vaccine technologies, such as viral-like particle and nanoparticle vaccines, DNA/RNA vaccines, and rational vaccine design, offer innovative approaches to address existing challenges of vaccine development. They have also significantly advanced our understanding of vaccine immunology and can guide future vaccine development for many diseases, including rapidly emerging infectious diseases, such as COVID-19, and diseases that have not traditionally been addressed by vaccination, such as cancers and substance abuse. This review provides an integrative discussion of new non-viral vaccine development technologies and their use to address the most fundamental and ongoing challenges of vaccine development.

## Introduction

Beginning with the discovery of cowpox inoculation that can protect humans against smallpox infection by Edward Jenner in the late 18^th^ century, vaccination has become an important means to prevent disease. Despite the success of vaccination in the eradication or control of some major pathogens, several challenges remain in vaccine development and administration. Several widespread infectious diseases such as HIV, tuberculosis, and influenza continue to pose great challenges for fully protective vaccination. Emerging and reemerging pathogens present a pressing need for expediting vaccine development and approval as a rapid response to epidemics, such as the current COVID-19 global pandemic caused by the SARS-CoV-2 virus. The advantages and disadvantages of the various vaccine platforms can make the choice for preferred platform(s) to use for vaccine development during a pandemic complicated. The traditional methods to produce a vaccine, such as live attenuated and inactivated vaccines or protein subunit vaccines have their advantages and disadvantages, which have been extensively reviewed elsewhere (1–3). Briefly, live attenuated vaccines present the risk of reversion to a highly pathogenic form while inactivated vaccines may not be sufficiently immunogenic or in some cases can lead to an enhanced disease pathology (3). Additionally, most pandemic vaccines have to be clinically tested during an active outbreak in order to obtain sufficient safety and efficacy data, thereby limiting the number of vaccine candidates that can be deployed to save life during an emergency situation.

Less conventional approaches to vaccinology include the non-viral vaccine technologies that are the topic of this review, as well as viral vector platforms. Viral vector vaccines rely on antigen delivered on an unrelated, non-pathogenic viral backbone. This technology was developed almost forty years ago using a vaccinia virus vector expressing the hepatitis B surface antigen (HBsAg), which provided protective immunity to chimpanzees exposed to hepatitis B (4, 5). Since then, viral vectors have been used successfully in many veterinary species (6–12), although only a single viral vector has been licensed for human vaccination (rVSV-ZEBOV for Ebola virus) (13). A number of viruses have been developed as vectors for vaccine development, including poxviruses, adenoviruses, herpesviruses, arenaviruses, retroviruses, paramyxoviruses, and flaviviruses, among others (14–16). The main advantage of viral vectors over traditional vaccines is their ability to evoke a robust adaptive immune response in the absence of an adjuvant (17). However, the tradeoff for enhanced immunogenicity is the concern for potential reversion of the attenuated viral vector to virulence, especially when using a replication-competent vector (18). Replication-defective and single-cycle viral vectors are attractive alternatives that have an increased safety profile and, in some cases, are still able to elicit a strong immune response (19, 20). More details about the known viral vectors and their recent advances in vaccine development will be discussed in our forthcoming review article (Vrba, S.M., et al., in preparation).

Other fundamental challenges toward successful vaccination include the ever-changing and highly divergent nature of some viruses that allow for the potential to escape vaccine coverage, pre-existing immunity of the vaccinated populations, and pre-existing medical conditions that can prevent vaccines from being fully effective and safe. Vaccination could also provide an enticing alternative therapy against diseases such as cancers and substance abuse. However, the efficacy of these vaccines is limited by the disease complexity and the lack of a more complete understanding of protective immunity in these medical conditions. The relative contribution and balance of the different arms of host immunity, i.e., antibodies and cell-mediated responses, toward protection without adverse effects remains a challenging issue that needs to be addressed for individual disease (21). Furthermore, the immune response to vaccination can be influenced by numerous factors such as gender, age, co-existing medical conditions, genetic variations, and lifestyle (22). While vaccines have traditionally been delivered as inactivated or attenuated preparations, recent developments of non-viral vaccine systems offer potential additional solutions to meet the new challenges of vaccine development, especially during epidemic or pandemic situations. This article focuses on new non-viral vaccine development technologies and their implications for combating on-going and emerging communicable and non-communicable diseases.

## Emerging Technologies in Non-Viral Vaccine Development

### Virus-Like Particle and Nanoparticle Subunit Vaccines

Subunit vaccines deliver antigens as purified proteins, which confer the advantage of enhanced safety and scalability compared to whole-pathogen vaccines due to the lack of the requirement for the expression of all viral components and the ability to express and purify any particular antigens of interest in large quantity. A disadvantage of subunit vaccines is that they are generally less immunogenic in nature and therefore require adjuvants and repeated vaccination doses (2). Several approaches have been used to increase the immunogenicity and stability of subunit vaccines, such as virus-like particle (VLP) vaccines and nanoparticle (NP) vaccines.

**Figure 1:**
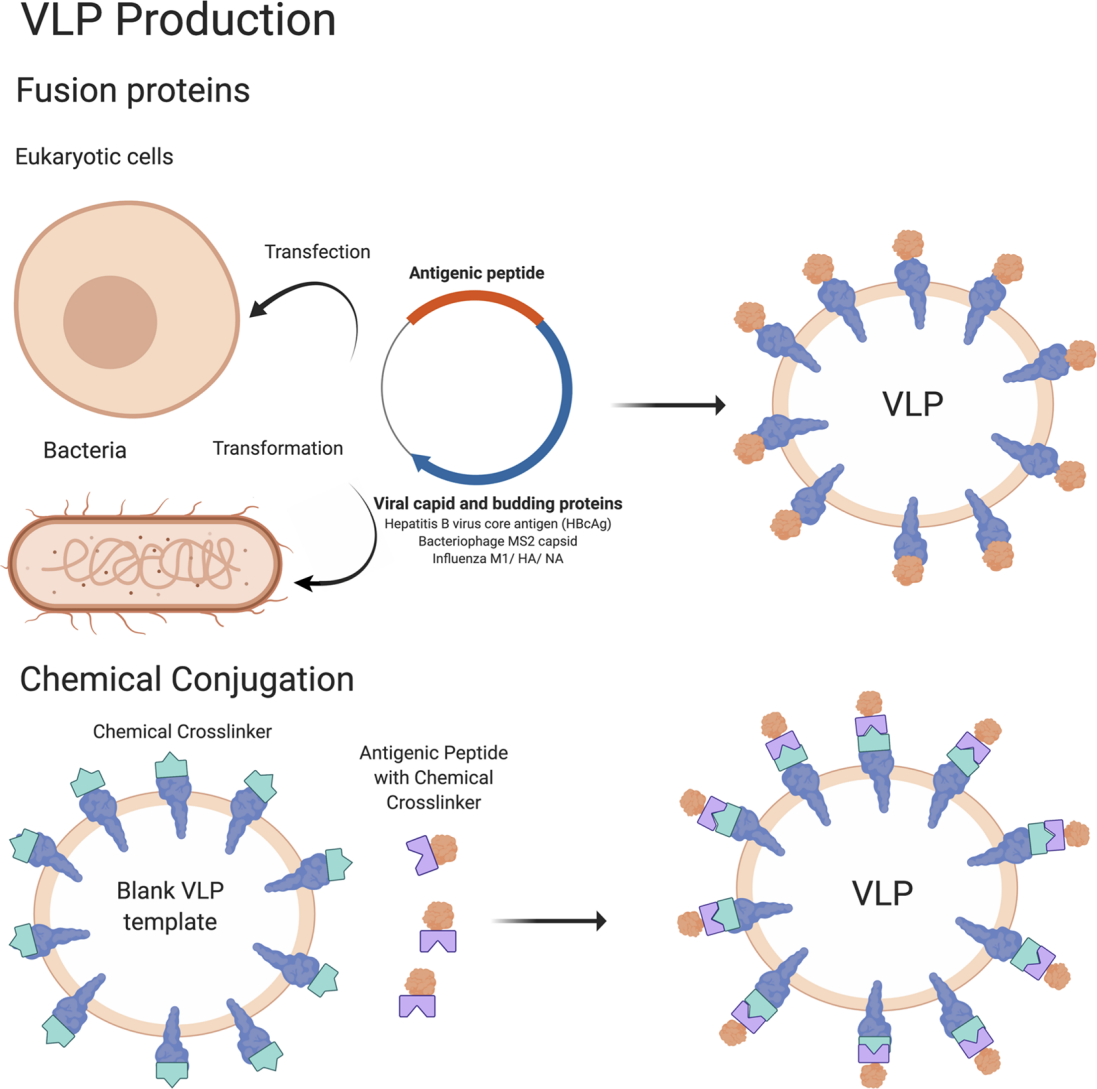
Schematic of VLP vaccine production. The methodology to produce VLP vaccines is summarized in this cartoon. In brief, VLP vaccines are produced by transfecting eukaryotic cells or transforming bacterial cells with a DNA plasmid encoding an antigenic peptide attached to a viral capsid and/or other protein that is sufficient to form VLPs. The antigenic peptide is present on the outside of the VLP which becomes available for interaction with the immune system. Antigens conjugated with a chemical crosslinker can also be attached to VLPs containing external proteins conjugated to a complementary chemical crosslinker, which will result in antigens being linked to the VLP and being presented on the outside edges. Figure created using BioRender software.

VLP vaccines use platforms capable of producing particles that mimic the structure of authentic viruses. VLP vaccines can be produced by expressing antigenic proteins in a eukaryotic or prokaryotic system, resulting in the formation of particles with an inherent ability of the antigenic proteins to self-assemble (23) **(** **Figure 1** **)**. Alternatively, VLP vaccines can also be made by producing blank VLP templates and then chemically linking antigenic peptides onto the pre-formed particles (23). Because these VLPs do not contain a viral genome, they are unable to replicate in cells and therefore have an improved safety profile compared to live viral vaccines (24). Yet, VLP vaccines can often fully activate immune systems of the vaccinated individual. VLPs are taken up by dendritic cells, where they are processed and presented on MHC class I and II molecules to activate the adaptive immune response. Subsequent stimulation of CD8^+^ T cells and CD4^+^ T helper cells leads to activation of cell mediated responses and B cells (and antibody production), respectively (23, 25–29). As a result, VLP vaccines are considered highly immunogenic and can stimulate robust cellular and humoral immune responses due to their highly repetitive display of antigenic epitopes (30, 31). A number of VLP vaccine candidates are now clinically applicable with some notable examples including the hepatitis B vaccine (HBV) Engerix (32), the human papillomavirus vaccine (HPV) Cervarix (33) from GlaxoSmithKline (GSK), the HBV vaccine Recombivax^®^ (34), and the human papillomavirus (HPV) vaccine Gardasil^®^ (35) from Merck & Co, Inc. VLP vaccines that are currently in clinical trials include vaccines for malaria (36, 37), influenza (38), rotavirus (39, 40), tuberculosis (41), Zika virus (42), and HIV (43, 44). Efforts to further increase the immunogenicity of VLP vaccines include optimizing antigen design and production platforms (of primarily bacterial origin) (45).

**Figure 2:**
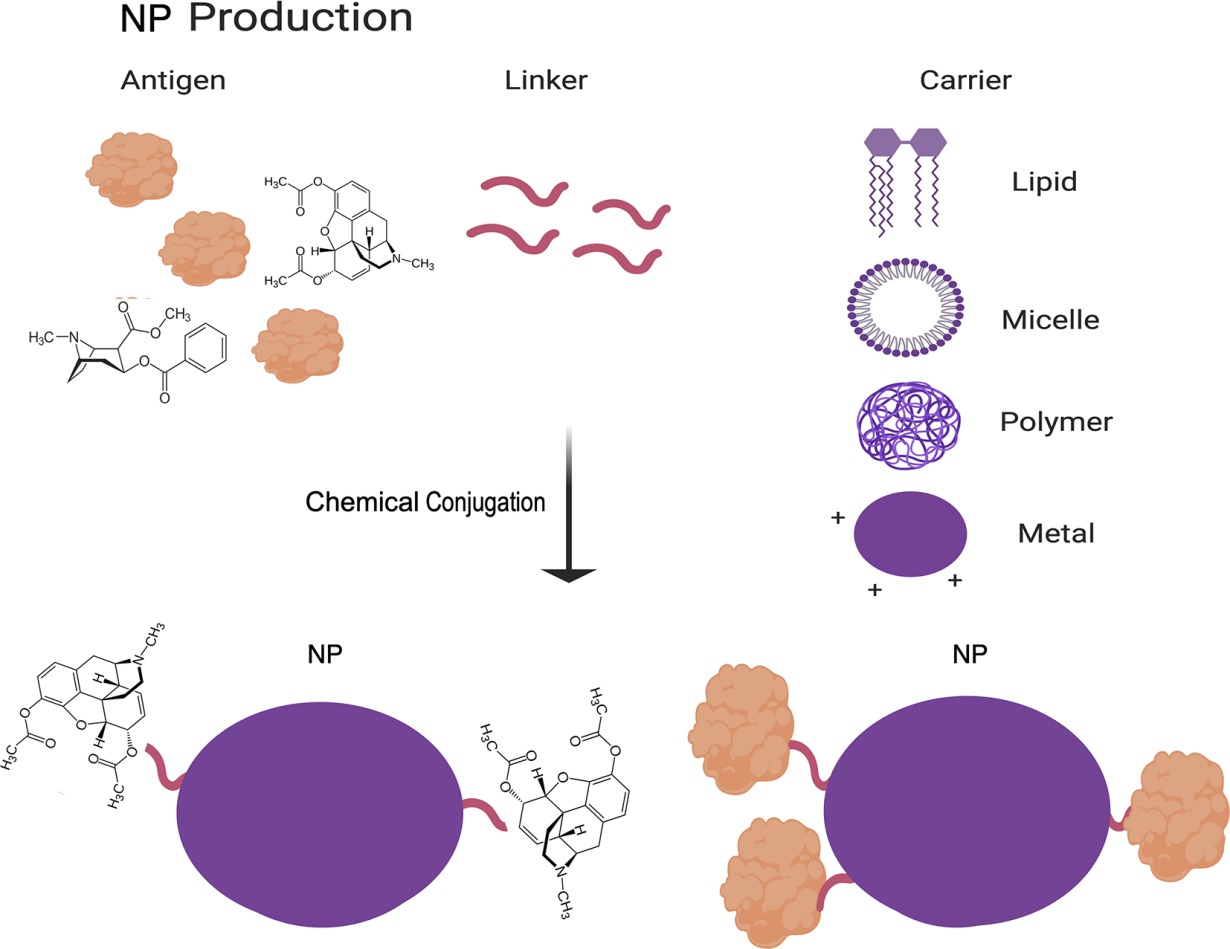
Schematic of NP vaccine production. The methodology to produce NP vaccines is summarized in this cartoon. In brief, NP vaccines are produced by assembling a complex of antigens, a linker molecule, and a carrier molecule by chemical conjugation. Figure created using BioRender software.

NP vaccines are produced by chemically crosslinking protein antigens and carrier molecules to increase immunogenicity and decrease degradation of the antigens (45). These carriers can be organic (primarily lipid-based) or in-organic (primarily polymeric or metal based) (**Figure 2**) (46–49). More recently, self-assembling protein NPs, which consist of oligomers of monomeric protein, have been found in some cases to also provide the benefit normally afforded by a carrier (47, 50). NPs have similarly high rates of stability as VLPs, but they do not stimulate the innate immune response to the same extent as VLPs. However, NPs are simpler in design than VLPs by lacking the multiple protein components of VLPs, which further decrease their cost of production and increase their reproducibility and safety. The challenges associated with decreased immunogenicity of NP vaccines as compared to VLP vaccines can be partly addressed by adjusting the carrier to the desired antigen, based on factors such as size, surface charge, shape and hydrophobicity (46, 47, 50). Additionally, carriers can be used to directly target NPs to immune cells and to increase cross-presentation by antigen-presenting cells (APCs) (46, 47).

### DNA and RNA Vaccines

Another promising area of vaccine development includes vaccines that are based on nucleic acids: DNA or RNA vaccines. These vaccines have gained popularity due to their cost-effectiveness, ease of design and production, attractive biosafety profile, and, in the case of DNA, stability. Nucleic acid vaccines have gained particular attention for their potential to rapidly produce vaccines against emerging infectious diseases such as those currently being tested against SARS-CoV-2, the causative agent of COVID-19, which will be discussed in some detail below.

**Figure 3:**
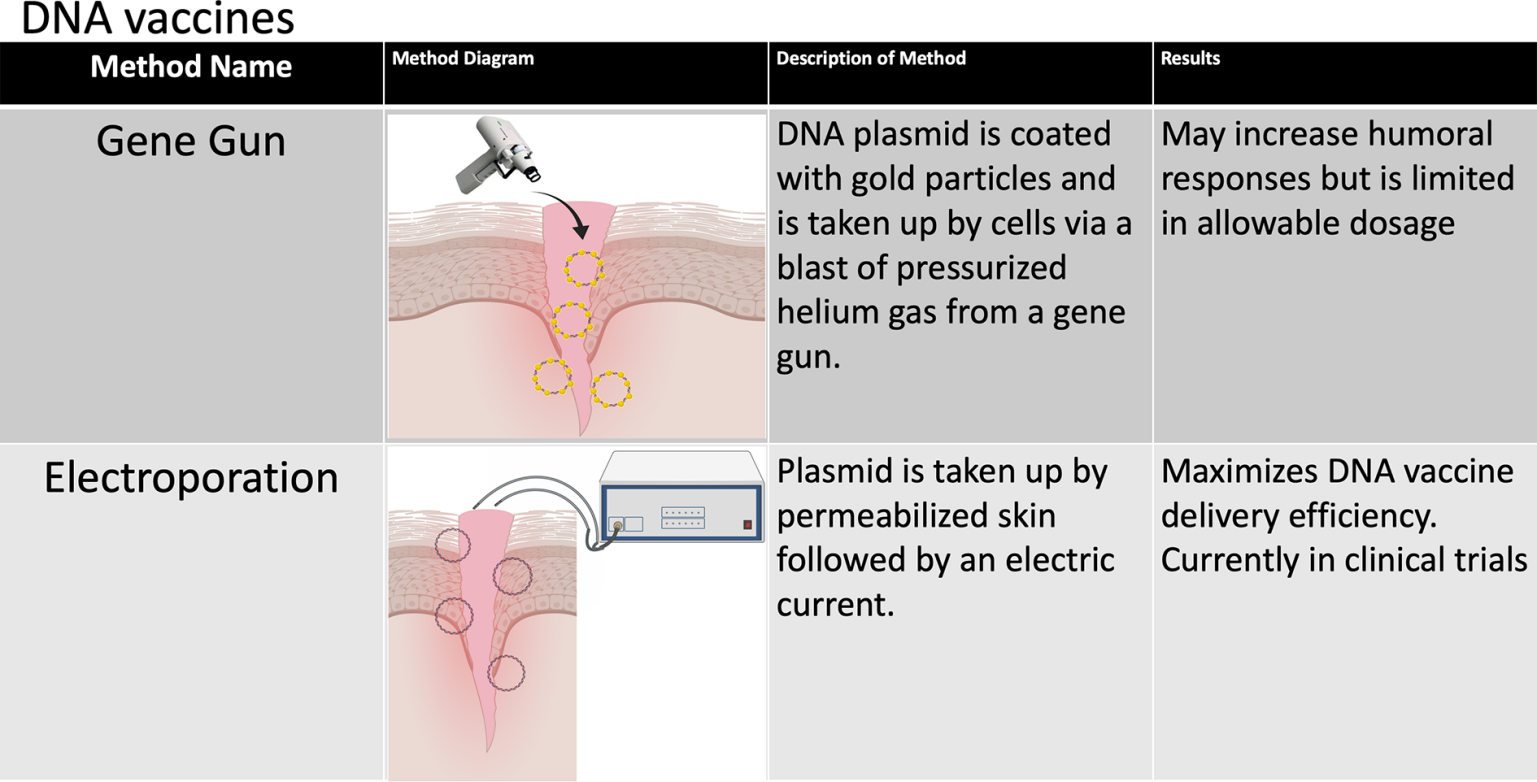
Methods of improving DNA vaccines. The various methods that have been developed to improve the stability and immunogenicity of DNA vaccines are summarized in this chart. A number of design and delivery mechanisms have contributed to improving the performance of nucleic acid vaccines, such as methods of clinical delivery, genetic engineering, and linking nucleic acid vaccines to cells or biomolecules. Figure created using BioRender software.

The immunogenic and protective efficacy of DNA vaccines have been demonstrated repeatedly *in vitro* and in small animal models, and a limited number of DNA vaccines have been approved for veterinary use (51). However, DNA vaccines tend to induce poor immune responses in humans and other large animal models (52). One possible explanation may be that intramuscular injection, which has been the most studied route of DNA vaccine administration in humans, tends to elicit mostly cell-mediated immune responses (53), which is likely due to significantly lower APC populations residing in muscles and antigen presentation dominated by MHC I (51). Alternatively, DNA vaccines can be coated with gold NPs and administered intradermally by a gene gun (**Figure 3**). While preliminary data suggest that this method may increase humoral responses to DNA-based vaccines (51), it is limited by its low dose per administration (54). In vivo electroporation (permeabilization of the skin by an electric current to allow plasmid DNA uptake) has thus far been shown to have the highest immunogenicity in multiple small animal models (51, 54) and has been tested in two phase I clinical trials for HIV vaccination with some promising results. In the first clinical trial, the immune system was primed with a DNA vaccine encoding the IL12 gene followed by a boost dose with the recombinant VSV-based HIV vaccine (55). The second trial evaluated the cellular immunity induced by HIV DNA vaccines through intramuscular injection administered by electroporation (56). Other efforts are being undertaken to increase the immunogenicity of DNA vaccines such as codon optimization, optimal promoter usage and epigenetic design, generating nanocarrier plasmids to increase stability and plasmids fused to proteins that specifically target APCs, adjuvant use (which will be discussed in some detail below) and short hairpin RNA (shRNA) targeting of host cells that decrease immunogenicity to DNA vaccines. These approaches have been extensively reviewed elsewhere (51, 54).

**Figure 4:**
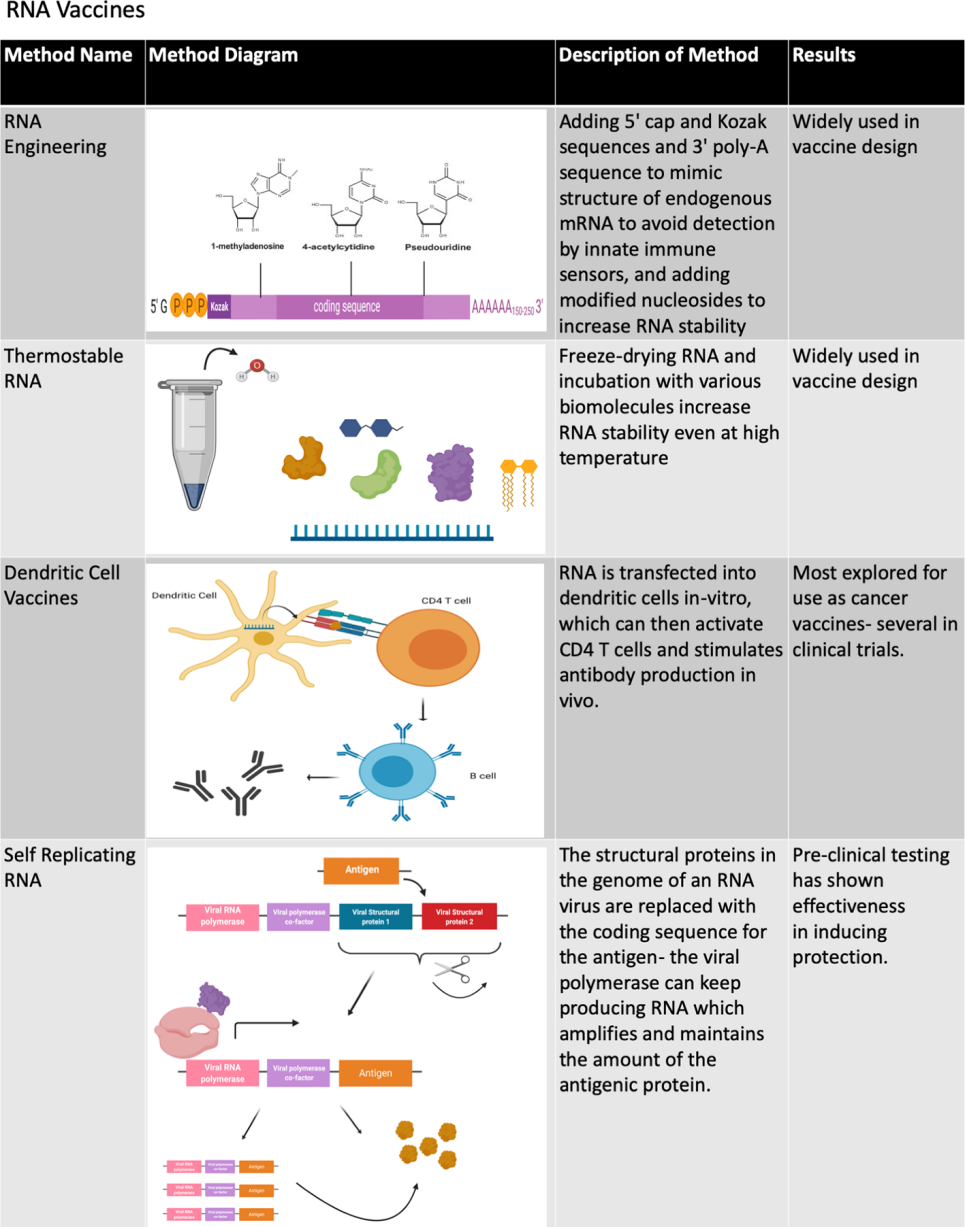
Methods of improving RNA vaccines. The various methods that have been developed to improve the stability and immunogenicity of RNA vaccines are summarized in this chart. A number of design and delivery mechanisms have contributed to improving the performance of nucleic acid vaccines, such as methods of clinical delivery, genetic engineering, and linking nucleic acid vaccines to cells or biomolecules. Figure created using BioRender software.

**Figure 5:**
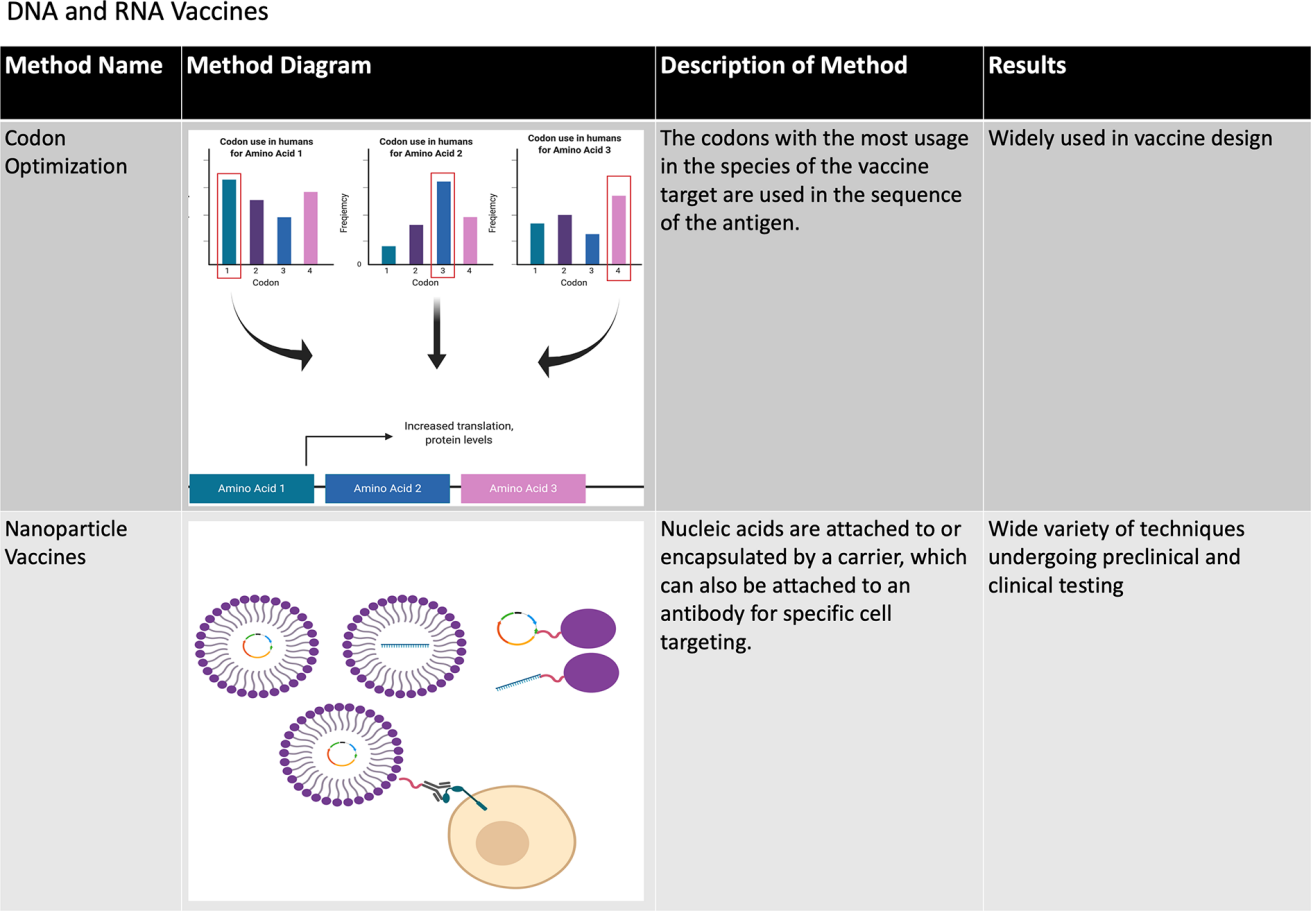
Methods of improving DNA and RNA vaccines. The various methods that have been developed to improve the stability and immunogenicity of both DNA and RNA vaccines are summarized in this chart. A number of design and delivery mechanisms have contributed to improving the performance of nucleic acid vaccines, such as methods of clinical delivery, genetic engineering, and linking nucleic acid vaccines to cells or biomolecules. Figure created using BioRender software.

A recent development involves the successful use of mRNA as a protective vaccine. While mRNA was originally found to be viable for *in vivo* gene transfer in the early 1990’s, the development of mRNA vaccines was initiated much later due to the inherent instability of mRNA compared to DNA (57). The efficacy of mRNA vaccines can be increased by several factors, such as ensuring mRNA purity, adding 5’ Kozak and cap sequences, 3’ poly-A sequences and modified nucleosides to increase mRNA stability and decrease detection by the receptors of innate immune cells, codon optimization, introduction by intramuscular, and intradermal injection to reduce RNA degradation, and by generating thermostable mRNA (57–59) (**Figures 4**, **5**). Methods to encapsulate RNA have also been explored to increase the stability and immunogenicity of RNA vaccines, as has been used with exosome encapsulated RNA (60) and RNA-transfected dendritic cells (61, 62). When fully optimized, RNA vaccines may have an immunogenic advantage over DNA vaccines due to the presence of multiple cellular pathways that activate innate immunity in response to foreign RNA such as the toll-like receptors (TLRs) and RIG-I-like receptors (RLRs) (63, 64).

In addition to the aforementioned non-replicating RNA, RNA vaccines can include self-replicating or self-amplifying RNA molecules that are normally based on positive-strand RNA viruses of which the structural genes are replaced by antigens (57, 58) (**Figure 4**). One study comparing the efficacy of conventional mRNA versus self-amplifying RNA found that both were effective in protecting mice against influenza infection, but that self-amplifying RNA elicited protection at a much lower RNA dose and induced a delayed yet longer-lasting antigen expression (65). Self-replicating RNA transfected into dendritic cells (66) has been shown to induce an immune response *in vivo* (67). RNA vaccines have been used in a number of studies in animal models (68) and have recently completed phase 1 clinical trials for rabies (57, 69) and influenza (68, 70). Both trials had similar safety profiles with most patients experiencing mild to moderate reactions to administration and a few patients experiencing more severe reactions. Both vaccines also demonstrated immunogenicity through neutralizing antibody levels, though antibody levels with the rabies vaccine were more highly dependent on dose and route, with needle-free intradermal dose able to sustain neutralizing antibody levels in half of the number of vaccinated individuals one year after injection but not with those receiving intramuscular or intradermal injections (57, 69). Additionally, phase 1 clinical trials are currently underway to test the self-replicating RNA vaccines for HIV and Zika virus (68).

Several challenges face the development of DNA/RNA vaccines. First, while DNA and RNA vaccines may avoid the safety concerns due to microorganism-based vaccine formulations, they have safety concerns of their own. While an early study suggested that DNA vaccination might result in some instances of random chromosomal integration, it was determined that this occurred with a significantly lower frequency than random genetic mutations (71). However, a subsequent study did not observe chromosomal integration to occur following DNA vaccination (51). The possibility of introducing unwanted bacterial DNA elements (such as antibiotic resistance genes to the gut microbiome) has been raised as a safety concern for DNA vaccination, but as of yet it has not been proven (51). As such, regulatory guidelines have been put in place for new DNA vaccine clinical trials in the United States and Europe (72). Vaccine formulation based on mRNA has the advantage of being produced in cell-free systems that can eliminate the concern of bacterial contamination and also lack the potential for chromosomal integration and long-lasting expression (57). While the World Health Organization (WHO) has recently classified mRNA vaccines as its own therapeutic class (73), similar regulations have not yet been developed due to the more limited testing of mRNA vaccines in humans.

It has been found that DNA vaccination primarily induces antigen expression at the site of administration with significantly lower levels being observed elsewhere (51), which may partly explain its poor immunogenicity. While less is known about the levels of on- and/or off-target expressions seen with RNA vaccination, they are presumed to be generally lower than DNA vaccine due to the decreased stability of RNA. However, off-target antigen expression may be relatively minor in shaping the immune response, as the route and mode of DNA/RNA vaccine delivery can markedly alter vaccine immunity, but the mechanism is yet to be fully understood. Generally, intramuscular or intradermal injection is used in animal models and human volunteers to elicit protection against infectious disease to maximize delivery to APCs, while intraperitoneal or intravenous injection has been used in selected animal models to induce systemic expression in therapeutic models, such as cancer vaccination (57). These findings implicating localized dosage routes as most effective for eliciting immunity from nucleic acid vaccines may help explain why gene gun and electroporation have been found to be the most effective routes for DNA vaccine administration. The most effective dosage routes may also be similar for RNA vaccines, as intradermal and intramuscular injection have repeatedly been found to be the most effective delivery routes for RNA vaccines, and needle-free delivery systems may also be more effective than injections (57). Interestingly, immunity can still result from injection of naked RNA in certain models, but it has been found in particular that IV administration of naked RNA results in rapid RNA degradation (57).

### Rationally Designed Vaccines

A key aspect of non-viral vaccine development involves the selection of antigens that can effectively elicit a protective immune response. Whereas traditional vaccines are generally developed through attenuation or inactivation of pathogens and through the incorporation of few selected antigens as vaccine components, new technologies have recently been applied toward antigen discovery and design. For example, “reverse vaccinology” refers to the ability to screen the complete antigen sets based on whole-genome sequencing of pathogens for the ability to induce protective antibody responses. Combinations of reverse vaccinology and traditional vaccine approaches allow for an efficient development of immunogenic vaccine candidates (74, 75).

Bioinformatic tools have contributed greatly to vaccine design and evaluation in recent years. Computational approaches are continually improving in their ability to predict T and B-cell epitopes from the complete antigen pools and to rationally design antigens with potential long-lasting protective immunity. Such algorithms calculate antigen-antibody interaction energies and structures (76) that increasingly bridge modeling based on existing templates and free-modeling based on heterologous structures and database consensus design (77). Deep sequencing combined with computational analysis allows for thoroughly characterizing the B cell repertoire in survivors of disease to identify the protective immunity (78).

A computationally designed antigen found to be protective in animal models was first reported for the respiratory syncytial virus (RSV) F antigen in 2013 (79). Many challenging vaccine targets have since been developed rationally for HIV (80–82), hepatitis C (83, 84), herpes (85), Zika (86, 87), RSV (82), HPV (88), as well as for bacteria (82, 89), fungi (90), and cancers (91). Rational vaccine design has also been utilized to improve VLP and NP vaccines by selecting a repetitive and predictive protein backbone structure for enhanced antigen presentation (92). Finally, rational antigen design is being explored for activation of dendritic cells (93) such as targeting C-type lectin receptors to activate antigen presentation in the context of the pathogens (e.g., Ebola and HIV) (94). The first rationally-designed vaccine to undergo human clinical trials is the anti-malarial vaccine Mosquirix, which was approved for use by the European Medicines Agency in 2015 (95). Human clinical trials have not yet begun for other rationally designed vaccines, however. A key point to note is that rationally designed vaccines require a comprehensive knowledge of the biology and immune response to a pathogen (95), and rational design is therefore more difficult to implement for rapidly emerging diseases.

A major challenge to rational vaccine antigen design is the lack of knowledge of T cell epitopes compared to B cell epitopes. Most successful antigens are expected to elicit both B and T cell responses. Quantitative databases have been developed more for predicting B cell receptor (BCR) epitopes than T cell receptor (TCR) epitopes. BCR epitopes can be predicted in part by the structural and chemical properties of the epitopes due to BCRs recognizing primary and tertiary antigen structures, while TCR epitopes have to be predicted based on known TCR epitopes because they only recognize the primary structures. Increasing capacity for identification of TCR epitopes by machine learning from known epitopes will likely help to mitigate this inequity (74, 75, 96).

## Non-Viral Vaccine Systems to Address Ongoing Challenges of Vaccine Development

### Vaccines for Immunosuppressed Individuals

A fundamental challenge to vaccination is the limited immunogenic response to vaccines seen in immunosuppressed individuals, namely, young children, the elderly, and those who are immunocompromised for medical reasons. The underlying causes of immunosuppression in each of these populations vary, and their underlying mechanisms should be taken into account when creating the best vaccine approach.

Young children, in particular infants and neonates, are considered to be immunosuppressed due to age-specific immune system developments that result in children being particularly susceptible to infection (97). The specific mechanisms for the immunosuppression in this population vary, but one prominent example is the decreased expression of Th17 supporting cytokines by TLR receptors and increased expression of anti-inflammatory cytokines in neonates and particularly in premature newborns (98). On the other hand, the immunosuppressive phenomenon observed in the elderly has been referred to as immunosenescence (99) and it is caused by a number of complex changes resulting in impaired innate and adaptive immune responses (100–104), degradation of lymphoid architecture (105), and increasing proinflammatory cytokines and chemokines (106, 107). To highlight a few important changes, dendritic cells have reduced uptake of antigens (108, 109), macrophages are unable to phagocytose apoptotic cells (110), the number of naïve T cells decreases (111, 112), and B cell repertoire decreases (113). These age-related changes in the ability of the immune system to respond to infection differ from the challenges to vaccination presented by conditions or medications that result in immunosuppression. One such example of a medication that results in immunosuppression is steroids, which have been reviewed extensively elsewhere (114). Steroids exert many effects on immune cells, such as the reprogramming of dendritic cells to tolerogenic dendritic cells (115–117). These cells induce the formation of regulatory T cells (118).

The development of vaccines that can potently activate the innate immune response without using live attenuated vaccines is a central focus of vaccine development for immunocompromised individuals. This is particularly relevant for subunit vaccines, as they do not contain potential viral genomic elements that can act as pattern-associated molecular patterns (PAMPs) to activate innate immune responses. A major approach toward increasing the immunogenic response to a subunit vaccine is through the use of adjuvants. Originally discovered by including food products in equine vaccines and inducing sites of localized sterile inflammation and abscesses, the adjuvant repertoire has since been greatly expanded (119). The so-called “first generation adjuvants”, which remain the most common adjuvants in clinical use, include aluminum salts (alum) and mineral oil-in-water emulsions, which function by promoting the migration of APCs to the sites of intramuscular injection (120). However, the use of these adjuvants is limited greatly by the recruitment of only a comparatively small population of immune cells that are made up of APCs (120) and a markedly Th2 response with little to no cellular immune response (120). This has become the aim of current research to design new adjuvants that can increase the breadth of the innate immune responses to the vaccine.

Much effort has been focused on enhancing the usable adjuvant repertoire to further customize the immune response and to avoid the Th2-dominated immune response seen with some adjuvants (e.g., alum) and instead support a Th1 response in certain circumstances. Specifically, a response skewed toward Th2 response is desired for antibody production and antiparasitic immune response, while a Th1 response is desired for intracellular or viral pathogens. Skewing toward Th1 or Th2 is thought to occur after APCs stimulate certain cytokine gene expression profiles (121). For example, LPS-derivative–based AS04 is being used in hepatitis B and HPV vaccines (122) and has been found to increase cell-mediated immune responses in patients with end-stage renal disease (123). Other adjuvants that have been developed to induce a Th1 immune response include IC31^®^ (124, 125), GLA-SE (125, 126), and CAF01 (125, 127). In addition to the Th1 skewed immune response that these adjuvants displayed, GLA-SE induced antibodies and CAF01 showed a Th1/Th17 response (125). Increasing the breadth of the immune response to vaccines thus can enhance the safety and effectiveness of vaccines for both immunosuppressed populations as well as the general population.

There has been an increasing effort toward developing new vaccines that may induce a safe and immunogenic response in immunocompromised individuals. As an example, DNA vaccines could be used to encode for antibodies that could be safely and temporarily expressed in immunocompromised patients, such as throughout the course of an influenza season. Recent studies that tested the efficacy of influenza neutralizing antibodies found that protection against lethal disease could be conferred by plasmids expressing antibodies given intramuscularly by electroporation (128). However, several major considerations need to be fully addressed before these techniques can be developed for human use. Specifically, the duration and the stability of plasmid vaccination have not yet been fully characterized in humans. Additionally, it has been shown that anti-dsDNA antibodies can be produced by primary B cells isolated from mice treated with plasmid DNA (129), which appear similar to anti-dsDNA antibodies that have been shown to be expressed during systemic lupus erythematosus (130, 131). The anti-dsDNA antibodies may prefer to bind to certain CpG-rich sequences on bacterial DNA of the plasmid (129), which might serve as a means for DNA vaccine optimization. Finally, the purity of plasmid DNA stocks needs to be thoroughly confirmed in order to avoid possible stimulation of unwanted immune reactions.

Other novel adjuvant approaches include the surfactant and emulsifier-based AS03 that are currently being used in influenza-pandemic vaccines (132). LPS-derivative–based AS04 is being used in hepatitis B and HPV vaccines (122) and has been found to increase cell-mediated immune responses in patients with end-stage renal disease (123). Lipid products that form micelles in solution and act as solid particle carriers are another form of adjuvants that can activate innate immunity, as seen with CAF01 (133, 134) and AS01B/E formulation from GlaxoSmithKline (135), which are used in the only currently available vaccine for malaria (136, 137). Several other adjuvants currently in use primarily function as TLR agonists (138–140). TLR agonists have shown promise in aged and young mice (141) as various TLR agonists [e.g., CpG (TLR9), poly(I:C) (TLR3), and pam3CSK4 (TLR1 and TLR2)] can induce expression of co-stimulatory molecules on APCs (141). Another adjuvant approach taken to overcome the immune challenges presented by young children is the use of ß-glucan. These sugars, found in the cell walls of some pathogens, activate dendritic cells through the CLEC71-SYK-CARD9 pathway, and it was shown to provide protection against tuberculosis infection (142). Recently, defective-interfering (DI) viral particle vaccines have also been explored for use as adjuvants to increase the innate immune response (143–152). These are VLPs with aberrant and defective genomes, which have been found in some cases to increase the innate immune response when compared to the replicating virion. Taken together, several innovative strategies are currently being developed to increase the immunogenicity and safety of vaccines for immunocompromised populations.

### Vaccines With Non-Traditional Antigens

Because of their increased safety and versatility, such as the ability to deliver a diverse range of molecules as antigens, VLP and NP vaccines have the potential for use to provide immunity against non-protein antigens. A prime example is the development of NP vaccines to treat substance abuse disorders by attaching a drug molecule to a hapten carrier (**Figure 2** **).** Vaccines against drugs of abuse aim to elicit a humoral (antibody) response that can neutralize the drug target before it crosses the blood-brain barrier to induce psychotropic effects, thereby decreasing positive associations with and hopefully dependency on the addictive drug. Such vaccines have an advantage for long-term therapeutic use over pharmaceuticals targeting neural receptors by eliminating the medical complications and safety concerns of directly modulating neural signaling network. They also differ from other vaccines in that they are given to active users of drugs of abuse to prevent escalation of use or relapse and do not depend on herd immunity for effectiveness, so their efficacy is determined by individual responses to the vaccines (153).

A hapten carrier, which is a potent B cell antigen, is used to stimulate B cell responses and thereby also activates B cell responses to the attached drug. Therefore, hapten and linker design are of particular interest to ensure structural integrity and to maximize B cell responses (153–170). The vaccine can also be linked to a protein carrier designed to activate T cell responses (and particularly CD4 T cell response to then activate B cells) (153, 160, 171–181), though there has not been a clear determination of whether an increased CD4 Th2:Th1 ratio correlates with efficacy for vaccines against drugs of abuse (153). Finally, an additional consideration in designing vaccines against drugs of abuse is determining whether to target the drug itself or its possibly more psychotropic metabolites that can provide a greater level of protection. A prime example of this is heroin vaccines seemingly being most effective when they can structurally mimic the psychotropic heroin metabolite 6-acetylmorphine (6AM) (166, 172, 182). It should be noted that clinical trial results have only been reported for vaccines against nicotine and cocaine addictions, with the vaccines demonstrating efficacy only in a subset of patients that were able to achieve high neutralizing antibody titers (153, 183–186). The recent vaccine developments to ameliorate drug abuse have been reviewed more extensively elsewhere (153, 160, 187).

Additionally, VLP and NP vaccines are being used in toxoid vaccine formulations, which provide quick neutralization against a cytotoxic molecule (primarily bacterial toxins) that cannot be expressed in its full and activated form. The most well-known example is the diphtheria, tetanus and pertussis (DTaP) inactivated subunit vaccine which has been in use for decades and can elicit effective immune responses against the toxin produced by any of these bacteria if/when the vaccinated individual happens to be exposed to them. The pertussis component of DTaP has a demonstrated high level of safety that it is one of only two known vaccines (besides influenza) that is given to pregnant women in several countries (188). Current clinical trials are focusing on testing potentially more effective toxoid vaccines for pertussis (189) as well as *Haemophilius influenzae* type b, polio virus, and hepatitis B virus (190). It has also been found that using bacterial membrane or red blood cell (RBC) membrane micelles as a carrier can significantly increase the immunogenicity of the vaccines, and pre-clinical testing is currently underway to use these carriers in vaccine development against the multi-drug resistant bacteria *Staphylococcus aureus* (MRSA) (191).

### Therapeutic Vaccines for Noncommunicable Diseases

As our capabilities for vaccine development and production have expanded, a paradigm shift has recently taken place to use vaccines for disease treatment in addition to disease prevention. These therapeutic vaccine designs rely on the identification of protein markers unique to a disease phenotype which may evade the development of an immune response due to the markers not being recognized by the APCs. For example, cancer vaccines to elicit immune responses against cancer-specific antigens are one of the most widely studied therapeutic vaccines to date due to the inherent challenges of developing effective cancer therapeutics and a need for targeted treatment. The immunosuppressive environment present in cancers has made developing cancer vaccines a significant challenge, especially for vaccines that rely on viral vectors. Therefore, the improved safety profile of non-viral vaccines offers an attractive potential for cancer vaccine development. Non-viral vaccines also confer an additional advantage for developing cancer vaccines in that cancer vaccines may be most effective when an antigen specific to the mutational profile of the individual cancer is used (192–194), particularly in combination to overcome immune tolerance (195). The time needed to make a vaccine against an individual antigen or against a combination of individual antigens is greatly reduced with non-viral vaccines due to the ease of encoding an antigen on a nucleic acid vaccine or purifying protein for a subunit vaccine in comparison to incorporating a personalized antigen into a viral vaccine, growing viral stocks and verifying its expression (196).

Nucleic acid vaccines have been a key area in recent developments for cancer treatment. While a number of DNA cancer vaccine candidates have entered into early phase clinical trials (197), RNA vaccines are thought to have particularly encouraging potential due to their increased immunogenicity compared to DNA vaccines (68, 198). Preliminary results indicate that intranodal injection of naked tumor antigen-encoding mRNA can control tumor growth in mouse cancer models (199–202). Additionally, naked mRNA was found to be immunogenic *via* intradermal injection in a phase I/II clinical trial for prostate cancer (203). However, a key challenge in developing RNA cancer vaccines has been the need to further ensure the stability of the RNA and to increase its targeting to APCs (204, 205) in order to overcome the immunosuppressive environment of cancers. Developing a delivery vehicle for RNA cancer vaccines has therefore been a central focus of research and development in this area.

Loading RNA into liposomes is one method that has shown some success in controlling cancer growth in mouse models (206–208) and has demonstrated some preliminary efficacy in early stage clinical trials for use as a delivery system for anti-cancer genes (209, 210) and siRNAs (211), with different liposome constructs targeting RNA localization to the spleen. RNA-loaded liposomes can also be targeted directly to T cells by using RNA that encodes for anti-CD3 along with the cancer antigens, bypassing the need for recognition by APCs. This concept has notably been tested in conjunction with chimeric antigen receptor (CAR) T cell therapy (198, 212, 213). Another method bypasses targeting RNA to APCs by directly transfecting dendritic cells (DCs) with RNA extracted from tumors or RNA encoding tumor antigens (214–217) **(** **Figure 4** **)** and then introducing the engineered DCs into patients with a combination of cytokines and/or checkpoint blockades (218, 219). However, DC-directed RNA vaccines are currently limited by the restrained immune environment present in cancers, which can limit the activity of DCs and increases the activity of regulatory T cells (216, 220, 221). It is thought that these challenges could be mitigated by optimizing the use of cytokines and other factors that would act as adjuvants in combination with cancer RNA vaccines (195, 222–225) and by optimizing DC isolation and culturing conditions (226). A few DC-directed RNA vaccine candidates are currently in clinical trials, including those in phase III (196, 221).

Subunit vaccines have also been developed for use as cancer vaccines (227), which are being tested with many of the same delivery systems as nucleic acid-based cancer vaccines to maximize vaccine targeting to immune cells (228). NP-based vaccines in particular have been developed and tested for use as cancer treatments (228–235), the most notable of which are several HPV vaccines for prevention of cervical cancer (236). While less development has been done on VLP-based cancer vaccines, one notable target that has been used is the widely expressed cancer antigen human epidermal growth factor receptor-2/neu (HER2), which has shown to be immunogenic in mouse models (237–244) and in early clinical testing in humans (245) and dogs (246), but as a whole these vaccines have had to undergo additional design in order to overcome B cell tolerance (227, 247) and to fully characterize their anti-tumor activity.

Because subunit vaccines require antigen presentation in order to elicit an immune response, a primary challenge in VLP- and NP-based cancer vaccine developments has been optimizing their uptake by APCs (228). Vaccine uptake by APCs can be optimized by engineering VLP- and NP-based cancer vaccines to resemble the structure of viral particles as closely as possible, such as by using certain types of carriers (liposomes, polymers and ferritin cages), sizes (20–45 nm) and a spherical shape **(** **Figure 4** **).** Subsequently, these vaccines may be most successful when combined with checkpoint blockade treatment by encouraging clonal expansion of lymphocytes (248). VLPs and NPs can also be used as immuno-enhancers, e.g., to deliver cytokines and TLR agonists to target sites, which has been found to boost localized immune responses while avoiding immunopathogenic and possibly systemic inflammation (46).

### Vaccines for Rapidly Emerging Viral Diseases

Emerging and reemerging pathogens, such as West Nile virus, pandemic influenza virus, Ebola virus, dengue virus, Zika virus, and the on-going global pandemic SARS-CoV-2 pose great challenges to the public health system. Rapid development and deployment of vaccines are critical to quickly build up resistance against these and other disease “X”, which is a term used by the WHO to refer to future unknown disease pandemics (249). The ideal vaccine platform in a pandemic situation must be cost-effective and can be rapidly developed and produced on a large scale to meet global demands. Temperature sensitivity is also a consideration, as cold chain storage can be particularly difficult to maintain in developing countries. Development of heat stable vaccines like the oral bovine rotavirus pentavalent vaccine (BRV-PV, Rotasil^®^ by the Serum Institute of India), which was prequalified by the WHO in 2018, can provide protection against serious diseases in regions where transportation and refrigeration are unreliable (250). In comparison, the only FDA approved Ebola virus vaccine (rVSV-ZEBOV, ERVEBO^®^ by Merck and Co., Inc.) must be stored at −80°C or −60°C (251), which presents a major obstacle for affected countries. Rapid production of low cost, scalable, and temperature stable vaccines is an ongoing challenge in the face of emerged and emerging global disease pandemics.

Currently, rapid development of vaccines is greatly limited by the resources and regulatory policies needed to bring a vaccine from its conceptualization stage to the clinic, which has been estimated to cost between $200 and $500 million dollars and to take 5–18 years (252). Vaccines also tend to be manufactured in countries with larger economic and technical prowess and more robust disease surveillance systems than developing countries and therefore can unfairly influence the equity of vaccine distribution and usage. This was seen in the 2009–2010 influenza pandemic, where 80% of the vaccines were manufactured and used in seven industrialized regions (United States, Canada, Australia, western Europe, Russia, China, and Japan), while the majority of developing regions in the world did not receive any pandemic influenza vaccines until January 2010, 9 months after the WHO declared the influenza pandemic (253). In addition, as mentioned previously, most pandemic vaccines have to be clinically tested during an active outbreak in order to obtain sufficient safety and efficacy data, thereby limiting the number of vaccine candidates that can be deployed to save lives. This was seen during the Ebola outbreak of 2013–2015, when two vaccines were fully developed in advance of clinical trials but only one (the simian adenovirus-based Ebola vaccine ChAd3-EBO-Z) was tested early enough in the outbreak to obtain sufficient clinical data (254). Similar challenges are also seen in selecting vaccine candidates for the large sample sizes needed for phase III clinical trials for HIV vaccine candidates. Statistical ranking systems to prioritize candidates are being developed to aid in this selection process (255). Zoonotic diseases present additional considerations, as it is economical to vaccinate the multiple species that may act as reservoirs of the pathogen(s) in order to control the spread of the disease. The first vaccine to provide protection in multiple species is the simian adenovirus-based vaccine candidate ChAdOx1 RVF, which has been shown to provide effective protection against Rift Valley Fever virus in sheep, goats, and cattle and is currently undergoing testing in larger livestock field trials and in humans (254, 256).

Other measures have been undertaken to expedite the process of vaccine development and reduce the cost of vaccine production. International institutions allow for collaborative groups to rapidly co-operate on vaccine development and shorten the vaccine manufacturing process. The Coalition for Epidemic Preparedness and Innovations (CEPI) provides funds for clinical trial and stockpiling of vaccines that would not have market incentive in a traditional funding mechanism of vaccine development and manufacturing (257). Such international collaborations will help to bridge the differing vaccine development policies and investitures across countries and use these combined resources to develop vaccines to primarily benefit those living in either underdeveloped or developing nations (258). In a recent example of this, CEPI, Gavi, and the WHO have come together to form COVAX, the vaccines pillar of the Access to COVID-19 Tools (ACT) Accelerator, with the mission to expedite the production of a COVID-19 vaccine to be equitably distributed throughout the world (259).

Technical challenges in vaccine production process can be an impediment. For example, the use of fertilized chicken eggs in vaccine production can pose challenges such as the restricted capacity of egg production, egg allergies, and the emergence of viruses with egg-culture-adapted mutations that can reduce vaccine efficacy (260). The use of animal cells for certain vaccines can also present significant challenges of cost, slow production rates, and potential high risk of contaminations. Other vaccine production systems, such as VLP vaccines produced in yeasts, insect cells and bacterial systems, as well as DNA/RNA vaccines, can benefit from increased robustness of antigen production, decreased risk of contaminations, and quicker time of response (252). This may especially be the case for DNA vaccines, where the increasing capacity of next-generation DNA sequencing, for example, has lowered the time for development of a DNA vaccine from 20 months following the 2003 SARS outbreak to 3.25 months following the 2016 Zika outbreak (261).

### COVID-19 Pandemic as a Case Study to Rapidly Develop Non-Viral Vaccines

The ongoing COVID-19 pandemic has presented unique opportunities as well as challenges for vaccine development. Unlike the influenza vaccines, no coronavirus vaccines existed prior to the COVID-19 pandemic. Such a rapid and widespread need for a completely novel vaccine for COVID-19 has resulted in a drive to significantly reduce the length of time required to produce a new vaccine. It has also highlighted the necessity to use non-viral vaccine platforms with overlapping stages of vaccine development, including preclinical and clinical testing and manufacturing that would otherwise be required to happen in a stepwise process for a traditional vaccine development effort (262–264). However, a rapid progression of clinical testing will need to be balanced by the need for obtaining quality data on vaccine safety and efficacy (265), especially considering previous reports of pathological antibody-dependent enhancement responses in some patients immunized with the 2003 SARS vaccine candidates (266, 267). As with previous pandemics, widespread global availability and resource management will be another key consideration for vaccine selection, especially considering the near ubiquitous presence of COVID-19 around the globe and its disproportionate impact on populations of low socio-economic status (268, 269). It is also likely that the approval of multiple vaccine candidates will be most optimal to controlling and ending the pandemic should more than one vaccine prove to be effective in preventing COVID-19 disease. Multiple COVID-19 vaccines would allow for more clinical and regulatory choices to accommodate differences in patient responses (particularly in more vulnerable patient populations) and manufacturing and distribution capabilities (270, 271). Perhaps, with these considerations in mind, non-viral COVID-19 vaccine platforms (e.g., DNA and mRNA) have been selected among the first candidates to enter clinical testing, partly for their aforementioned reasons of safety profiles and relative ease of manufacturing **(** **Table 1** **).** Some of the RNA-based COVID-19 vaccines (all of which are currently in various stages of clinical trials) include but are not necessarily limited to:

1. The mRNA-1273 vaccine developed by the U.S. biotech company Moderna (272).
2. The mRNA CVnCoV vaccine developed by the German company CureVac (273).
3. A group of 4 RNA vaccines under the name BNT162 developed by the German company Biontech that consists of two nucleoside-modified mRNAs, a uridine-containing mRNA and a self-amplifying mRNA (274), which in an early phase I/II trial, the nucleoside-modified mRNA BNT1621b has been shown to elicit neutralizing antibodies (275) and is better tolerated particularly in older adults than BNT1621a (276, 277).
4. The self-amplifying mRNA LNP-nCoVsaRNA (COVAC1) vaccine from the Imperial College London (278).
5. The mRNA vaccine LUNAR-COV19 (ARCT-021) from US company Arcturus Therapeutics (279).
6. An unnamed mRNA vaccine candidate from Chinese company Yunnan Walvax Biotechnology (280).

**Table 1:** Non-viral vaccines currently in development for SARS-CoV-2*.

Vaccine Name	Vaccine type	Company and Country	Preliminary results
mRNA-1273	mRNA	Moderna, USA	Self-reported preliminary data indicating all patients developed neutralizing antibody response. Patients developed moderate side effects with highest dose (250 ug) were eliminated from future study. Entered phase III clinical trials in July 2020 with targeted enrollment of 30,000 people
CVnCoV	mRNA	CureVac, Germany	Entered phase II clinical trials in August 2020
BNT162	mRNA (4 candidates)	Biontech, Germany	Early phase I/II trial data showed that patients who received nucleoside-modified mRNA BNT1621b produced neutralizing antibodies. Further phase I/II clinical trial data showed that BNT1621a and BNT1621b produced similar neutralizing antibody titers but that BNT1621b was associated with less systemic responses particularly in older adults. BNT162b was selected to continue in phase II/III clinical trials.
LNP-nCoVsaRNA (COVAC1)	mRNA (self-amplifying)	Imperial College London, UK	Entered phase I/II clinical trials in June 2020 Transitioned to phase II clinical trials in July 2020
LUNAR-COV19 (ARCT-021)	mRNA	Arcturus Therapeutics, USA	Entered phase I/II clinical trials in July 2020
Unnamed mRNA vaccine	mRNA	Yunnan Walvax Biotechnology co, China	
INO-4800	DNA	Inovio, USA	Preliminary phase I data suggest that 94% of participants developed an immune response against the vaccine. Preprint suggests that a single dose seroconverted vaccinated rhesus macaques. Neutralizing antibodies were produced against the D614 and G614 strains and memory responses lasted at least 4 months after vaccination.
GX-19	DNA	Genexine, South Korea	Entered phase I/II clinical trials in June 2020
AG0301-COVID19	DNA	AnGes Inc, Japan	Entered phase I/II clinical trials in July 2020
ZyCoV-D	DNA	Cadila Healthcare Ltd, India	Entered phase I/II clinical trials in July 2020
bacTRL-Spike	DNA (live bacteria delivery)	Symvivo, Canada	
LV-SMENP-DC	APC (lentiviral)	Shenzhen Geno-Immune Medical Institute, China	
Covid-19/aAPC	APC (lentiviral)	Shenzhen Geno-Immune Medical Institute, China	
AV-COVID-19	APC (antigen-loaded)	Aivita Biomedical, USA	Entered phase I/II clinical trials in May 2020
NVX-CoV2373	NP	Novavax, USA	Entered phase I/II clinical trials in May 2020 Self-reported data from phase I indicate that the vaccine was well tolerated and induced neutralizing antibody responses in all patients after two doses.
SCB-2019	NP	Clover Biopharmaceuticals, China	Entered phase I/II clinical trials in May 2020
COVAX-19	NP	GeneCure Biotechnologies, USA	Entered phase I clinical trials in June 2020
MVC-COV1901	NP	Medigen Vaccine Biologics corp, Taiwan	Entered phase I clinical trials in July 2020
AdmirSC-2f	NP	Adimmune corp, Taiwan	Entered phase I clinical trials in August 2020
Unnamed spike protein vaccine	NP	University of Queensland, Australia	Entered phase I clinical trials in June 2020
Unnamed VLP vaccine	VLP	Medicago, Canada	Entered phase I clinical trials in June 2020

*COVID19 vaccine data compiled with the aid of the BioRender COVID-19 Vaccine and Drug tracker: https://biorender.com/covid-vaccine-tracker.

This chart summarizes the name, type of vaccine, company and country of origin and preliminary data on existing non-viral vaccines for SARS-CoV-2 that are currently undergoing clinical testing.

Some of the COVID-19 DNA vaccines (all of which are also in various stages of clinical trials) include but are not necessarily limited to:

1. The INO-4800 vaccine developed by the U.S. pharmaceutical company Inovio (281) with preliminary phase I data suggesting that 94% of participants might have developed an immune response against it following vaccine administration by electroporation (282) and that vaccination in rhesus macaques elicited neutralizing antibodies against both the D614 and G614 SARS-CoV-2 strains (283).
2. The GX-19 vaccine developed by the South Korean company Genexine (284).
3. The AG0301-COVID19 vaccine developed by the Japanese company AnGes, Inc. (285, 286).
4. The ZyCoV-D vaccine developed by the Indian company Cadila Healthcare Ltd (287).
5. The live bacteria-mediated plasmid delivery system bacTRL-Spike developed by the Canadian company Symvivo (288).

Genetically engineered APCs are also being pursued as potential COVID-19 vaccine candidates, with DCs transfected with lentiviral vectors expressing COVID-19 antigens currently being tested in China (289, 290) and in the United States (291). Meanwhile, COVID-19 VLP- and NP-based vaccines have also advanced into clinical trials, including the NP NVX-CoV2373 vaccine from the U.S. company Novavax (292), the NP SCB-2019 vaccine from the Chinese company Clover Biopharmaceuticals (293), the NP COVAX-19 vaccine from the U.S. company GeneCure Biotechnologies (294), the NP vaccine from the Taiwanese company Medigen Vaccine Biologics (295), the NP vaccine AdmirSC-2f from Taiwanese company Adimmune corp (296), an unnamed NP vaccine from the University of Queensland (297, 298) and an unnamed VLP vaccine from the Canadian company Medicago (299). CEPI has collaborated in the development and testing of a selected number of these vaccine candidates (273, 281, 298).

It should also be noted that several viral vectored vaccine candidates for COVID-19 have also entered in clinical testing **(** **Table 2** **).** Several adenovirus vectored vaccines are currently the furthest along in clinical testing. One example is the vaccine candidate AZD1222 (formerly known as ChAdOx1 nCov-19), a replication-defective chimpanzee adenovirus developed by Oxford University which entered phase III clinical trials in August 2020. This viral vector was chosen due to its previous application as a vaccine vector for Middle East respiratory syndrome coronavirus (MERS-CoV). The ChAdOx1 vector encoding the spike (S) protein provided protection against six different strains of MERS-CoV in rhesus macaques (25), demonstrating its ability to be an effective vaccine for coronaviruses. Specific to COVID-19, AZ1222 was found to induce humoral and cell mediated immune repsonses in phase I/II cliical trial and did not result in any instances of severe side effects (300). It has recently found that AZD1222 could induce a robust humoral, CD8 and Th1 dominant CD4 response in mice and rhesus macaques and that both a prime and a prime-boost regimen protected rhesus macaques against COVID-19 related pneumonia. However, it should be noted that there was no difference in the amount of nasal virus shedding in vaccinated vs unvaccinated animals challenged with SARS-CoV-2 (301).

**Table 2:** Viral vaccines currently in development for SARS-CoV-2*.

Vaccine Name	Vaccine vector	Company and Country	Preliminary results
AZD1222 (ChAdOx1 nCoV-19)	Adenovirus	Oxford University, UK	Vector was shown to protect rhesus macaques against six strains of MERS-CoV. Early phase I/II clinical trial data show that vaccine was well tolerated and induced humoral and cell-mediated responses. Vaccine was found to induce robust humoral, CD8 and Th1 dominated CD4 responses in mice and rhesus macaques, and that both a prime and prime-boost regimen protected rhesus macaques against COVID-19 related pneumonia. Entered phase III clinical trials in August 2020
Ad5-nCoV	Adenovirus	CanSino biologics, China	Early phase I/II clinical trial data show that vaccine induced antibody and cell-mediated responses after a single dose and was well tolerated. Entered phase III clinical trials in August 2020 Approved by the Chinese government for use by its members of the armed forces
Ad26.COV2.S	Adenovirus	Johnson and Johnson, USA	Vaccine was found to induce antibody and T cell responses in rhesus macaques after a single dose, and antibody titers negatively correlated with viral titers during viral challenge. Entered phase III clinical trials in August 2020
Gam-COVID-Vac	Adenovirus	Gamaleya Research Institute of Epidemiology and Microbiology, Russia	Approved for widespread use by the Russian government before the release of clinical trial data Entered phase III clinical trials in August 2020
TMV-083	Measles	Institut Pasteur, France	Entered phase I clinical trials in August 2020
V591	Measles	Merck, USA	Entered phase I clinical trials in August 2020

*COVID19 vaccine data compiled with the aid of the BioRender COVID-19 Vaccine and Drug tracker: https://biorender.com/covid-vaccine-tracker.

This chart summarizes the name, viral vector used, company and country of origin, and preliminary data on existing viral vaccines for SARS-CoV-2 that are currently undergoing clinical testing.

Two other replication-incompetent adenoviral vectored vaccines for COVID-19 have also entered clinical trials. The Ad5-nCoV candidate from Chinese company CanSino biologics was shown in early clinical trial data to induce significant antibody and T cell responses after a single dose and to have only rare instances of severe side effects that were more prevalent among the higher dose groups (302, 303). The Chinese government has recently approved the vaccine for use among its members of the armed forces (304). Additionally, the Ad26.COV2.S from Johnson & Johnson induced antibody and T cell responses in rhesus macaques after a single dose, and antibody titers negatively correlated with viral titers during viral challenge (305). Finally, the Gam-COVID-Vac candidate from the Gamaleya Research Institute of Epidemiology and Microbiology in Russia is another adenovirus-based vaccine that is the first COVID-19 vaccine to gain government approval for widespread use after a phase I trial. Phase III trials for this vaccine began in August 2020 (306).

Finally, two COVID-19 vaccine candidates based on live-attenuated measles platforms have also entered into early clinical trials. The TMV-083 candidate from the Institut Pasteur and with collaboration with CEPI is a measles vectored vaccine expressing a modified SARS-CoV-2 surface glycoprotein that entered phase I clinical trials in August 2020 (307), while the V591 candidate from Merck also entered phase I clinical trials in August 2020 (308). Many other viral vectored vaccine candidates for COVID-19 are also in preclinical stages of development.

## Summary

The emergence of new non-viral vaccine technologies has significantly advanced the scope and efficacy of traditional vaccine formulations that are generally based on single protein subunit vaccines or attenuated or killed vaccines. Non-viral vaccine technologies have allowed for new applications to address ongoing challenges of vaccination with customization in the areas of safety, immunogenicity, breadth of protection, scalability, and ease of production. These new technologies have also expanded the notion of what is possible with vaccination by extending their reach to once untenable areas, such as cancer treatment and neutralization of drugs of abuse. It is clear that continued development and optimization of vaccines will require multi-faceted approaches that can only be implemented with extensive cross-field collaboration and periodic review of the current state of vaccinology. These challenges as well as opportunities ensure that vaccine development will remain on the cutting edge of science for decades to come to combat new and emerging pathogens as well as other noncommunicable diseases.

## Author Contributions

MB, SV, NK, and HL contributed to the literature review and writing of the manuscript. MB prepared all figures and tables. All authors contributed to the article and approved the submitted version.

## Funding

This work was supported in parts by NIH NIAID grant R01 AI131586, USDA-NIFA-Capacity Funds (Hatch and Animal Health), and the University of Minnesota School of Medicine Academic Investment Research Program (AIRP) and COVID-19 Rapid Response Funds to HL and YL, USDA-NIFA AFRI grant #2019-05384 and Minnesota Agricultural Experiment Station Rapid Agricultural Response Fund to HL, and by a pre-doctoral NIH fellowship T32 DA007097 to MB. NIH T32 training grant in Comparative Medicine and Pathology (5T32 OD010993-17) for NK.

## Conflict of Interest

The authors declare that the research was conducted in the absence of any commercial or financial relationships that could be construed as a potential conflict of interest.
